# Weighing the necessities and concerns of deprescribing among older ambulatory patients and primary care trainees: a qualitative study

**DOI:** 10.1186/s12875-023-02084-8

**Published:** 2023-06-30

**Authors:** Sheron Sir Loon Goh, Pauline Siew Mei Lai, Siti Nurkamilla Ramdzan, Kit Mun Tan

**Affiliations:** 1 Department of Primary Care Medicine, Faculty of Medicine, Universiti Malaya, Kuala Lumpur, 50603 Malaysia; 2 School of Medical and Life Sciences, Sunway University, Sunway City, Selangor 47500 Malaysia; 3 Division of Geriatric Medicine, Department of Medicine, Faculty of Medicine, Universiti Malaya, Kuala Lumpur, 50603 Malaysia

**Keywords:** Deprescribing, Older persons, Barriers, Facilitators

## Abstract

**Background:**

Deprescribing can be a challenging and complex process, particularly for early career doctors such as primary care trainees. To date, there is limited data from patients’ and doctors’ perspectives regarding the deprescribing of medications in older persons, particularly from developing countries. This study aimed to explore the necessities and concerns of deprescribing in older persons among older ambulatory patients and primary care trainees.

**Methods:**

A qualitative study was conducted among patients and primary care trainees (known henceforth as doctors). Patients aged ≥ 60 years, having ≥ 1 chronic disease and prescribed ≥ 5 medications and could communicate in either English or Malay were recruited. Doctors and patients were purposively sampled based on their stage of training as family medicine specialists and ethnicity, respectively. All interviews were audio-recorded and transcribed verbatim. A thematic approach was used to analyse data.

**Results:**

Twenty-four in-depth interviews (IDIs) with patients and four focus group discussions (FGDs) with 23 doctors were conducted. Four themes emerged: understanding the concept of deprescribing, the necessity to perform deprescribing, concerns regarding deprescribing and factors influencing deprescribing. Patients were receptive to the idea of deprescribing when the term was explained to them, whilst doctors had a good understanding of deprescribing. Both patients and doctors would deprescribe when the necessity outweighed their concerns. Factors that influenced deprescribing were doctor-patient rapport, health literacy among patients, external influences from carers and social media, and system challenges.

**Conclusion:**

Deprescribing was deemed necessary by both patients and doctors when there was a reason to do so. However, both doctors and patients were afraid to deprescribe as they ‘didn’t want to rock the boat’. Early-career doctors were reluctant to deprescribe as they felt compelled to continue medications that were initiated by another specialist. Doctors requested more training on how to deprescribe medications.

**Supplementary Information:**

The online version contains supplementary material available at 10.1186/s12875-023-02084-8.

## Background

Deprescribing is defined as “the process of withdrawal of an inappropriate medication, supervised by a healthcare professional with a goal of managing polypharmacy and improving outcomes” [1]. A previous systematic review found that approximately 20% of medications prescribed to older persons in primary care settings were potentially inappropriate [2]. Similar findings were found in a primary care clinic in Malaysia, where 21.3% of older persons had potentially inappropriate medications (PIMs) [3]. Hence, deprescribing potentially inappropriate medications (PIMs) could reduce harm and improve health in older persons [4].

Stopping unnecessary medications has long been recognized as part of good prescribing. In routine clinical practice, deprescribing is a challenging process [5] complicated by many factors related to patients and prescribers. To date, existing literature has only focused on a single stakeholder’s perspective, either from patients or doctors [6, 7]. However, successful deprescribing is likely to be dependent on both patients and doctors [8].

Previous studies on deprescribing medications in primary care settings were mainly conducted in North America, Europe, Asia and Australasia [4]. A search of published literature revealed that deprescribing is likely to be dependent on socio-cultural factors (such as familial and societal beliefs) [9] that are expected to differ between countries and even between healthcare settings within the same country. Cultural differences may affect patients’ attitudes about medical care, affect their ability to understand their health status and comprehend options for diagnoses and treatments [10]. When compared to their Western counterparts, Asians were found to be less participating in the decision-making process, whereby doctors are perceived as being more knowledgeable [11].

Primary care physicians are frequently the first point of medical contact and gatekeepers to specialist services [5]. However, there is limited data on primary care trainees’ views on deprescribing. During their early career years, primary care trainees started to develop good prescribing practices which could transfer into their later practice [12]. Hence, it may be worthwhile to capture their views which can help to develop an intervention to promote deprescribing. Besides, their views regarding deprescribing can offer insights into common practices and whether existing training programmes are adequate. Therefore, this study aimed to explore the necessities and concerns of deprescribing in older persons among older ambulatory patients and primary care trainees.

## Methods

This qualitative study was reported according to the Consolidated Criteria for Reporting Qualitative Research (COREQ) [13].

### Study design and setting

This qualitative study was conducted from July to December 2019 in a tertiary primary care clinic in Kuala Lumpur, Malaysia. In Malaysia, primary care services are typically delivered in two parallel systems: government-funded public primary care clinics and independently owned private primary care clinics [14]. Our setting was situated in a teaching hospital where we provide specialist training for primary care trainees in a government-funded primary care clinic.

In-depth interviews (IDIs) and focus group discussions (FGDs) were conducted among patients and doctors, respectively, to allow an in-depth exploration of deprescribing medication in older persons from the perspective of these two key stakeholders. To date, there is limited data on how to deprescribe medications for older persons in Malaysia. Hence, findings from this qualitative study could be used by researchers to develop an intervention [15, 16]. IDIs were conducted with patients to ensure the privacy of their personal health information, while FGDs were conducted for doctors to allow group dynamics for exchange of new ideas or insights that would likely emerge as a result of group exchange.

### Participants

#### Patients

Older persons (defined as individuals aged ≥ 60 years old), having ≥ 1 chronic disease, prescribed ≥ 5 medications and could communicate in either English or Malay were recruited. Patients were purposively recruited based on gender, age, and ethnicity (Malays, Chinese and Indians) to achieve maximal variation. Patients who had cognitive impairment or dementia were excluded.

#### Doctors

Primary care trainees (defined as doctors who are undergoing their 4-year specialist training in family medicine) were purposively sampled based on their stage of training (i.e., whether they were at years 2, 3 or 4, as this reflected the number of years of experience as a doctor). Year 1 primary care trainees were excluded as they were not practising onsite.

### Procedure

**Fig. 1:**
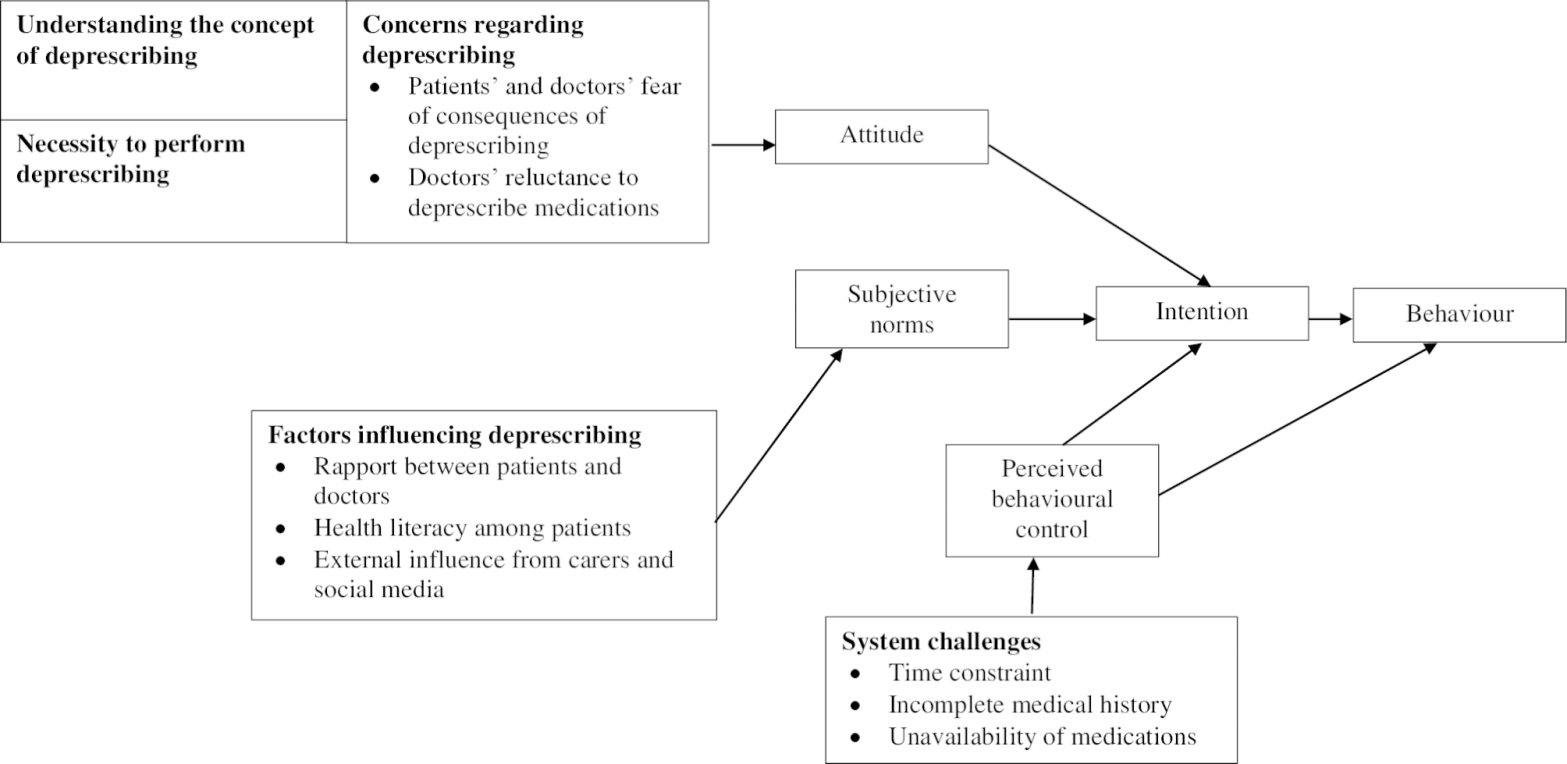
The conceptual framework of the necessities and concerns of deprescribing in older persons among older ambulatory patients and primary care trainees based on the theory of planned behaviour [19]

IDIs and FGDs were conducted using a semi-structured topic guide (Additional file 1) developed based on literature [6, 17, 18], Ajzen’s Theory of Planned Behavior [19] and expert opinion. The Theory of Planned Behavior was selected as the conceptual framework as it was widely used to postulate a person’s behaviour in performing a certain task [20]. This theory postulates that behaviour is influenced or determined by a person’s behavioural intention, which is influenced or determined by three independent variables: attitude, subjective norm, and perceived control (Fig. 1) [19]. The topic guide was pre-tested and minor changes to sentence phrasing were made. The pilot study findings were also included in the data analysis.

SSLG approached each patient and doctor individually at the clinic. The study’s purpose was explained to each potential participant. Participants were given the opportunity and time to ask any questions or request any information they needed about the study. Written consent was obtained from those who agreed to participate. A baseline demographic form was used to gather basic socio-demographic data prior to the IDIs and FGDs. All IDIs were conducted in a consultation room at the clinic in the patients’ preferred language (either English or Malay) by SSLG who is fluent in both languages. Each interview was audio recorded and lasted 30 to 60 min (median = 35 min).

All FGDs were conducted with the doctors and facilitated by SGSL (a trained researcher who was not an academician and therefore, would not be seen as an authoritative figure by participants) in the attendance of a note-taker. All FGDs were held in a meeting room. It was conducted in English, audio-recorded and supplemented by note-taking to indicate the order in which each participant spoke for accurate transcribing. Each FGD lasted 55 to 65 min (median = 60 min). No repeat interviews were carried out for both IDIs and FGDs.

### Data analysis

Both IDIs and FGDs were transcribed verbatim by experienced transcribers. All identifying information was removed from transcripts to maintain anonymity. SSLG then listened to the audio records to verify the accuracy of each transcript. Grammatical imperfections were retained to reflect the participants’ voices. Malay transcripts were analysed in the source language as researchers were fluent in both English and Malay. Malay quotations for this publication were translated into English using the forward and backward translation process.

Transcripts were analysed using the thematic approach, guided by the Necessity-Concerns Framework [21] and facilitated by Nvivo V.10 (QSR International Pty. Ltd., Victoria, Australia). According to the Necessity-Concerns Framework, key beliefs that influence medication adherence that may affect deprescribing are perceptions regarding the necessity and concerns of deprescribing [21].

SSLG immersed herself in the data by reading the first transcript line by line to develop the initial list of codes. The lists of codes were further refined and reduced in number by grouping them into themes and subthemes. This process was done interactively through consensus until the team (SSLG, PSML, SNR and KMT) agreed on the final coding framework. SSLG used the final coding framework for the remaining transcripts. The research team was consulted before any new codes were incorporated into the framework. Recruitment of participants ceased when data saturation was reached (defined to occur when no new themes emerged) [22]. The data for both IDIs for patients and FGDs for doctors were analysed as one data set using the same coding framework.

### Research rigour

Credibility, transferability, dependability, and confirmability were applied to the rigour evaluation criteria to ensure the trustworthiness of this study [23]. First, to enhance the credibility criterion, participants were recruited with maximal variation within our inclusion criteria, interviews were conducted using standardised topic guides (still allowing for adaption to the conversational flow) and participants were interviewed by trained researchers. Second, to ensure transferability, every detail of the data collection method, study period and participants was meticulously documented. Third, to ensure the dependability of data analysis, multiple experienced researchers with expertise in qualitative research were involved in data interpretation. Finally, to ensure confirmability, the researchers practised bracketing to ensure that their views on deprescribing medication for older persons did not influence how well they understood the participants’ experiences. This means that they separated their perceptions and participants’ experiences by acknowledging their biases and assumptions during data collection and analysis.

### Reflexivity

The research team consisted of two pharmacists (SSLG and PSML), a primary care physician (SNR) and a geriatrician (KMT). The research team (SSLG, PSML, SNR and KMT) had the knowledge and skills needed to conduct and analyse qualitative data. SSLG attended a workshop on “how to conduct qualitative research” and “how to use NVivo software to analyse the data”. PSML, SNR and KMT are experienced researchers in qualitative studies and have collectively published numerous qualitative research articles. All researchers were aware of their personal and professional biases on how to deprescribe medications in older persons. SSLG is a PhD student and registered pharmacist with a special interest in optimising medications in older persons. PSML is an academic pharmacist who advocates for medication safety in older persons. SNR is a primary care physician who advocates for patient-centred care and believes that discussions between primary care doctors and older patients about deprescribing should be initiated when a PIM is detected. KMT is a consultant geriatrician who is involved in the care and treatment of older persons and practices deprescribing in her daily clinical routine. Results were discussed iteratively within the team to ensure a balanced interpretation.

## Results

A total of 24/32 patients (response rate = 75.0%) agreed to participate (Table 1). Majority were male (n = 13, 54.2%) with a median age of 70 years. An equal number of Malays (n = 8, 33.3%), Chinese (n = 8, 33.3%) and Indians (n = 8, 33.3%) were recruited. Most patients completed secondary education (n = 11, 45.8%), The total median number of prescribed medications was six.

Meanwhile, 23/30 doctors (response rate = 76.7%) agreed to participate (Table 1). Majority were female (52.2%) with a median age of 33 years. The total median number of years of practice as a doctor was eight years. Four FGDs were conducted according to the stage of training (years 2–4) to facilitate the discussion as junior doctors could be intimidated by their seniors. The number of doctors participating in each FGD ranged from 5 to 6. Initially, one FGD was held for each training year (FGD1 = Year 4, FGD2 = Year 3, and FGD3 = Year 2). As new themes emerged in FGD2, another FGD was conducted to achieve thematic saturation. Based on the doctors’ schedules and availability, this FGD was conducted with a different set of Year 3 primary care trainees.

**Table 1:** Participants’ demographic details

No	ID	Ethnicity	Level of education	Number of prescribed medications	Group	ID	Years of practice
1. 2. 3. 4. 5. 6.	Patient01 Patient02 Patient03 Patient04 Patient05 Patient06	Indian Chinese Indian Indian Chinese Indian	Pre-university Tertiary Primary Secondary Secondary Secondary	8 5 8 9 7 6	FGD 1	Doctor01 Doctor02 Doctor03 Doctor04 Doctor05 Doctor06	13 9 10 10 9 7
7. 8. 9. 10. 11. 12.	Patient07 Patient08 Patient09 Patient10 Patient11 Patient12	Malay Indian Chinese Malay Indian Chinese	Tertiary Pre-university Pre-university Secondary Tertiary Pre-university	9 9 5 6 6 5	FGD 2	Doctor07 Doctor08 Doctor09 Doctor10 Doctor11 Doctor12	8 9 6 7 8 10
13. 14. 15. 16. 17.	Patient13 Patient14 Patient15 Patient16 Patient17	Indian Malay Chinese Indian Chinese	Secondary Pre-university Pre-university Secondary Secondary	5 6 5 7 8	FGD 3	Doctor13 Doctor14 Doctor15 Doctor16 Doctor17	7 7 10 6 8
18. 19. 20. 21. 22. 23. 24.	Patient18 Patient19 Patient20 Patient21 Patient22 Patient23 Patient24	Malay Malay Malay Chinese Malay Malay Chinese	Secondary Primary Secondary Secondary Primary Pre-university Secondary	7 8 5 6 6 5 7	FGD 4	Doctor18 Doctor19 Doctor20 Doctor21 Doctor22 Doctor23	7 7 8 8 7 9
	Median (IQR)				6 (5–8)		8 (7–9)

IQR = interquartile range

Four themes emerged from the data: understanding the concept of deprescribing, the necessity to perform deprescribing, concerns regarding deprescribing and factors influencing deprescribing (Table 2).

**Table 2:** Themes and subthemes on deprescribing

Themes	Sub-themes
Understanding the concept of deprescribing	-
Necessity to perform deprescribing	-
Concerns regarding deprescribing	a) Patients’ and doctors’ fear of consequences of deprescribing b) Doctors’ reluctance to deprescribe medications
Factors influencing deprescribing	a) Rapport between patients and doctors b) Health literacy among patients c) External influence from carers and social media d) System challenges i. Time constraint ii. Incomplete medical history iii. Unavailability of medications

### Understanding the concept of deprescribing

Patients and doctors understood the term ‘deprescribing’ differently, which affected their ability to explain what this term meant. Most doctors knew what ‘deprescribing’ meant, which is a process to stop or lower the dose or frequency of medication(s) that is no longer indicated or inappropriate. This process includes a comprehensive medication review on a patient’s medication list that could cause harm like unwanted adverse events. However, some doctors thought that deprescribing meant prescribing a ‘combination medicine’ (like Janumet®, which contains both sitagliptin and metformin) to reduce pill burden.

“…I think deprescribing means (to) stop prescribing (a) medication that is not necessary or causing harm…” [Doctor09/Year 3].

“I try to convert those medications with two or three times a day dosing to once a day dosing or to a combination medication…” [Doctor01/Year 4].

In contrast, most patients were unfamiliar with the term ‘deprescribing’. However, when this term was explained as ‘cutting down on the number of medication(s) you are currently taking’, most patients understood what deprescribing meant, and were receptive to the idea.

“If I’m feeling better, I want to cut down on these medicines. I don’t want to take too many medications” [Patient03].

### Necessity to perform deprescribing

**Fig. 2:**
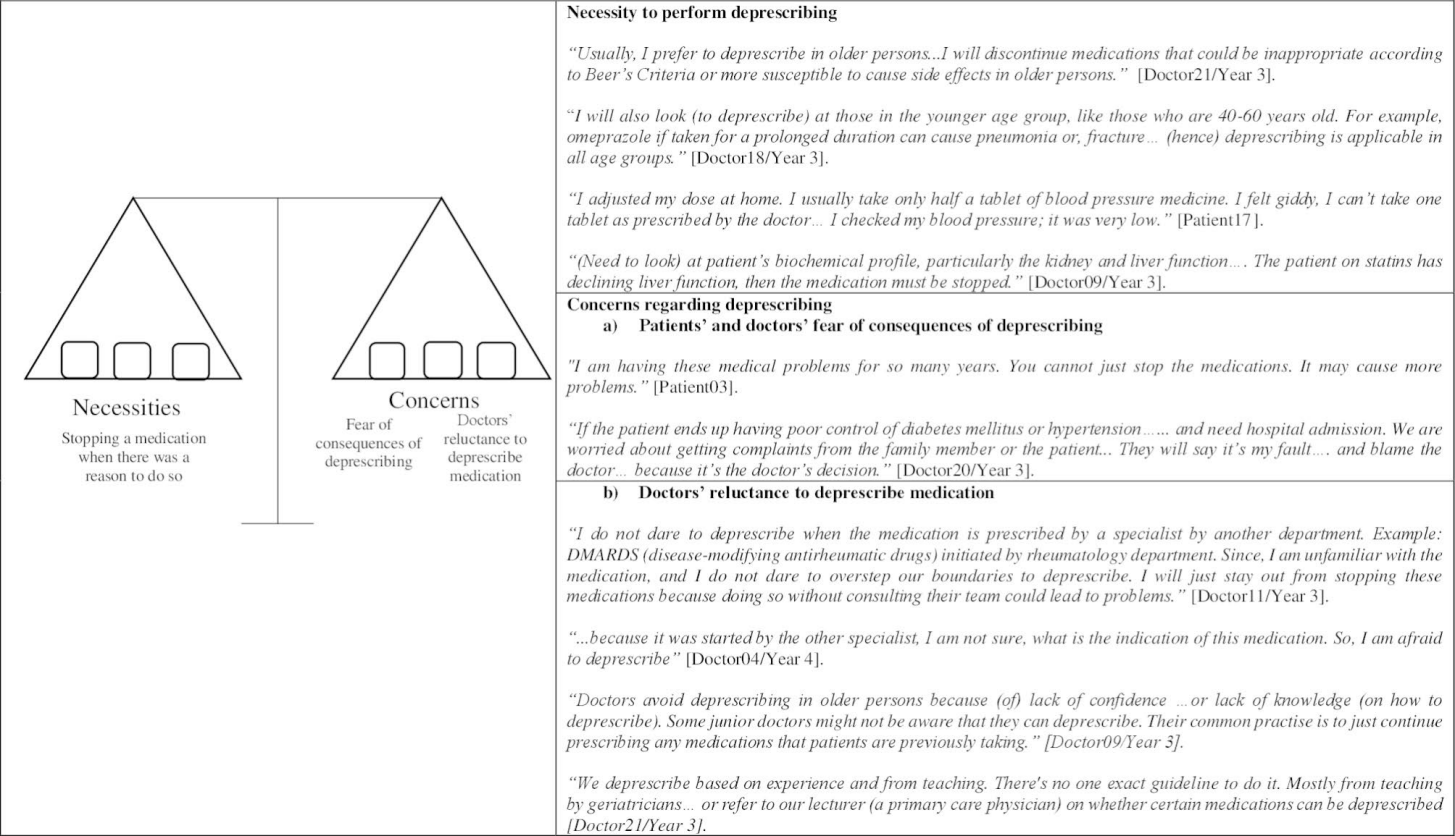
Necessities to perform deprescribing versus the concerns regarding deprescribing

Both doctors and patients thought that it was a necessity to deprescribe when there was an obvious reason to do so. Some doctors said that they would deprescribe in older persons, whilst others said they would consider deprescribing regardless of the patient’s age; especially if a PIM was detected. Other doctors would deprescribe medications causing unwanted adverse events. Likewise, some patients experienced unwanted adverse events and took less medication than instructed; and these were reasons why patients were keen on deprescribing. Doctors would also deprescribe medications in patients with declining kidney or liver function (Fig. 2).

### Concerns regarding deprescribing

However, both patients and doctors had a few concerns about deprescribing medications. There were two subthemes under this theme (a) patients’ and doctors’ fear of consequences of deprescribing and (b) doctors’ reluctance to deprescribe medications.

### Patients’ and doctors’ fear of consequences of deprescribing

Some doctors were reluctant to deprescribe patients who have stable conditions with their current medications, as they ‘did not want to rock the boat’. Doctors were afraid that older patients or carers would blame them when patients experienced unwanted adverse events (i.e. aspirin causing gastrointestinal bleeding if a proton pump inhibitor was discontinued) after the medication was discontinued. Patients also shared similar views stating that if they were doing well on their medications, they did not want anything changed (Fig. 2).

### Doctors’ reluctance to deprescribe medications

Some doctors felt compelled to continue medications that were initiated by another specialist from another department as they felt that some ‘specialized’ medical issues were beyond their expertise. The doctors were uncertain about the original prescriber’s intentions which made them reluctant to interfere with another specialist’s decision. Besides, some doctors reported that they lacked confidence and knowledge on how to deprescribe medications, which may lead them to deprescribe wrongly. Some doctors believed that continuing patients’ previous medications were easier because they did not need to perform a thorough medication review. Other doctors stated that if they encountered a difficult polypharmacy case, they would refer to a geriatrician or a primary care physician to deprescribe (Fig. 2).

### Factors influencing deprescribing

Four subthemes influenced participants to deprescribe (a) rapport between patients and doctors, (b) health literacy among patients, (c) external influence from carers and social media and (d) system challenges.

### Rapport between patients and doctors

When a PIM was identified, some doctors had no problems initiating a conversation with their patients about deprescribing medications. Before starting the deprescribing process, doctors gathered information about their patient’s experiences with their medications and discussed the deprescribing plan (including preferences and treatment goals) with them.

“I will assess whether deprescribing is possible for this patient. Then, I will discuss it with the patient. I will assess the patient’s reaction to my suggestion. Both patient and I must reach a mutually agreed decision before doing it.” [Doctor07/Year 3].

Some doctors reported that most patients preferred to follow a ‘doctor’s orders’ rather than actively participate in any deprescribing decisions. Some older patients had similar views as they felt that it was essential to take their medications as prescribed and feared their doctor will blame them for failing to do so.

“Maybe they are afraid to ask. They might think that they are trying to teach the doctor how to prescribe…the culture here, whatever the doctor says they just follow…” [Doctor04/Year 4].

“No choice. If I don’t take my medicines, I feel terrible and uneasy. The doctor will also scold me…” [Patient003].

Patients and doctors agreed that deprescribing required a long-term commitment from both parties and that this process could not be completed in a single visit. Doctors expressed that building a good patient-doctor rapport required continuity of care and trust; while patients thought that deprescribing would be easier if they were to see the same doctor for subsequent follow-up visits.

…sometimes the patient needs to have a rapport with us. Maybe this visit we cannot do it but we could introduce the patient to reduce the medication on the next visit. On the second visit, the patient is still not keen. Probably third or fourth visit, the patient will agree to it…” [Doctor12/Year 3].

“Doctors over here are on rotation. I don’t see the same doctor during my clinic visits. So, I am not sure whether the doctor can help me. If I see the same doctor regularly, I will know this doctor better, I will discuss it with him.” [Patient05].

However, some doctors faced difficulties in deprescribing medications to patients who did not speak the same language as they did. They were afraid that miscommunication could occur and the patient may not understand why changes were made to their medications.

“…Language barrier is part of the problem in multiracial Malaysia. If a patient comes alone, and cannot communicate well in Malay or English, I will find it very difficult to deprescribe. For example, if an Indian patient only understands Tamil, I will prefer the patient sees a doctor who can speaks Tamil…” [Doctor06/Year 4].

### Health literacy among patients

Some doctors reported they would be more willing to deprescribe in patients with higher health literacy. This was because doctors thought that these patients would have a better understanding of their health conditions and therefore be more capable to decide when to deprescribe medications. These patients would be able to perform blood pressure or blood glucose self-monitoring. Additionally, they would be able to recognize changes and understand the need to immediately see medical help if they become ill.

*“A patient is a good candidate for deprescribing when they perform home monitoring. When I stop that medication, the patient can monitor and if there is any problem, they will know and come back to see me immediately.”* [Doctor01/Year 4].

### External influences from carers and social media

Both patients and doctors stated that carers could influence their decision to deprescribe a medication. However, some patients preferred to make their own decision as they did not want to be misled by false information.

*“I think some patients are easily influenced. Even though I explained they can stop taking the medication and we decided to deprescribe it, I noticed they take back (restarted) the medication after a few months. This is because their friends or family members told them that the medication is important, and they must take it.“* [Doctor18/Year 3].

“The more people you ask; the more conflicting views you’ll get. That’s why I don’t listen at all and only stick to what doctors’ say.” [Patient09].

For patients who were unable to care for themselves, communication with a patient’s carer was vital during deprescribing. However, some patients were accompanied by different carers during their clinic visits. Hence, some doctors provided written instructions to carers to avoid miscommunication.

“An older person’s memory might not be that good. If they come with a carer, then it will help me to deprescribe… as the carer can provide me information about their medications…” [Doctor23/Year 3].

“The problem (occurs when) a different carer accompanies the patient to the clinic and the carer is a different person (not the main carer). So (I am) afraid my message doesn’t get delivered. I will write a memo to the (main) carer to inform the name of the medication that is stopped.” [Doctor05/Year 4].

Both patients and doctors reported that social media (like Google®, WhatsApp®) could be both beneficial and challenging. The availability of information at the fingertips has helped to ‘educate’ patients. However, some doctors were concerned that patients might obtain ‘false’ information regarding their medication from unreliable sources.

“Google. When they (doctors) give me any medicine I will check for medicine’s side effects.” [Patient24].

“Patients may get false information from social media. It’s quite difficult to convince them (to deprescribe) because they (patients) already read some information and do not agree with the information I am giving.” [Doctor19/Year 4].

### System challenges

System challenges that demote deprescribing were time constraint in clinics, incomplete patients’ medical history and unavailability of medications in healthcare facilities.

### Time constraint

Both patients and doctors reported that time was limited during patient-doctor consultations and deprescribing was time-consuming. A busy clinic environment was not conducive to initiate deprescribing.

“It’s very difficult to talk to them. Nowadays, doctors got no time to talk to patients. When I go in, at most she spends about five minutes.” [Patient21].

“We have very little time to talk to patients. I only have 15 minutes to see one patient. However, the deprescribing process takes up lots of time, especially in patients with multiple co-morbidities.” [Doctor03/Year 4].

Besides, some doctors admitted that they did not follow any deprescribing guidelines as they were lengthy and time-consuming to read. Instead, they preferred to have more continuous medical education programmes to improve their awareness and knowledge of deprescribing.

“We usually have no time to read guidelines. They are lengthy and occasionally difficult to read. I would suggest that more continuous medical education programmes on deprescribing should be introduced to improve our knowledge on deprescribing.” [Doctor21/Year 3].

Hence, some doctors suggested that doctors and pharmacists should collaborate to identify any PIMs. Pharmacists could assist doctors to conduct a comprehensive medication review, giving doctors more time to focus on other issues such as diagnosing and treating the patient. Some doctors suggested that an internal audit of the appropriateness of prescribed medications could be conducted by specialists as part of a hospital-level strategy for identifying PIMs. Audit, education and feedback regarding the deprescribing process can then be used to enhance their ability to deprescribe.

“If the patient has polypharmacy, we can refer to the pharmacists. They will check the patient’s medication one by one (perform a medication review). How the patient takes his/her medications and whether the patient knows about their medications.” [Doctor03/Year 4].

“Probably, an internal audit will help. When some of the cases were audited by lecturers (primary care physicians), they can point out which medication can be deprescribed.” [Doctor19/Year 3].

### Incomplete medical history

Doctors struggled to deprescribe when they were unable to identify medications that were brought to the clinic in unlabelled containers. To overcome this problem, some doctors suggested having a medication chart containing photos of certain medications at the clinic. Encouraging patients to bring their medications list was another option. A user-friendly electronic medical/health record (EMR) would also assist in the medication review process.

“Other facilitators is that patient brings along their medication list to clinic… At the place, I used to work, we have a medication chart with the shapes and names. So I can show and ask them how are they taking their medications.” [Doctor02/Year 4].

“The EMR itself, it’s not user-friendly. We take time to search back previous records if patients have multiple prescriptions from different clinics in this hospital. It will be good to have one master prescription…for us to see the entire list.” [Doctor22/Year 3].

#### Unavailability of medications

Doctors reported that they were reluctant to deprescribe or switch to a better medication if the medication was not available at another healthcare facility.

“Plavix® (clopidogrel) can be difficult to get from government health clinics. That’s why we are reluctant to change aspirin to Plavix® (clopidogrel) when patients complain of having dyspepsia or risk of gastrointestinal bleeding. We have no choice but to continue prescribing PPI (proton pump inhibitor) and inform patients about the long-term side effects” [Doctor11/Year 3].

Both patients and doctors reported that it was easier to deprescribe medications in patients who could not afford to purchase them.

“If they cannot afford that medication, I will review it. If it’s not necessary, I will just deprescribe.” [Doctor06/Year 4].

*“They ask me to buy… I say where am I getting the money from? Forget about it. Don’t want to take it.”* [Patient 04].

## Discussion

Four themes emerged from the data. Doctors had a good understanding of the deprescribing concept whilst patients were receptive to the idea when the term ‘deprescribing’ was explained. Both patients and doctors would consider the deprescribing of medications when the necessity outweighed their concerns. Factors that influenced the deprescribing process were rapport between patients and doctors, health literacy among patients, external influences from carers and social media and system challenges.

This study found that doctors had a good understanding of the deprescribing concept whilst patients were unaware of the term ‘deprescribing’ but were receptive to the idea when explained. These findings were similar to previous studies, whereby only 13% of medical students [24] and 7% of older adults recognised the term ‘deprescribing’ [25]. This may be due to the deprescribing concept being only introduced in 2003 [26]. Recognising the ‘term’ deprescribing is important as it may increase the acceptability and frequency of deprescribing conversations between patients and doctors [25]. Hence, integrating the term into daily clinical practice with patients and introducing deprescribing in medical education [27] could increase the awareness of deprescribing and the likelihood of initiating it [25].

This study found that both patients and doctors would only deprescribe medications when the necessities outweighed their concerns. Deprescribing medications are reported to be performed when a medication is no longer indicated, appropriate or aligned with evolving treatment goals [5, 28]. Similar to previous studies, the primary reasons to deprescribe among older persons are advancing age, unwanted adverse events and declining liver or kidney function [28]. The medication list for older persons lengthens with increasing age or when more comorbidities develop [28]. As a result, this will increase their risk to experience unwanted adverse events due to polypharmacy [28]. Besides, age-related physiological changes (like declining liver or kidney function) may have an impact on the pharmacokinetics and pharmacodynamics of medications, potentially altering the body’s reaction to them [29].

However, deprescribing was not performed when concerns outweighed necessities. Both patients and doctors were afraid of negative consequences like adverse effects due to medication withdrawal or disease relapse [6, 17]. They preferred to ‘maintain status quo’ in medically stable patients [30]. Doctors (in particular trainees) felt a reluctance to deprescribe, especially when a medication was started by another specialist [31–33], not knowing the original indication of the medication or feeling that the medical issue was beyond their scope of practice [34].

Building good rapport between patients and doctors for patient involvement and shared decision-making is important for successful deprescribing [35]. However, patients in Asia (like Malaysia), were found to be less participating in the decision-making process; and preferred their doctors to make decisions for them when compared to their Western counterparts [11]. This could be due to Asian patients’ tendency to view doctors as more knowledgeable [36] as they had a high level of trust in their doctors to decide for them [37]. Meanwhile, some patients perceived that their medications are highly important as they are essential for their overall well-being and stopping them would be harmful [37]. Therefore, a patient-centred approach should be considered before deprescribing so that patients and doctors could discuss decisions about medications, treatment goals and preferences [38, 39]. Besides, Malaysia is a multiracial country where many languages and dialects are spoken. This could make it more difficult for patients and their healthcare providers to communicate effectively about their health status and treatment options when compared to countries where only one language is spoken by the majority. Language barriers could result in poorer patient engagement, poorer quality of healthcare delivery, thus compromising patient safety [40]. Audio-visual aids, educational materials or utilizing carers as translators could be ways to overcome this problem [41, 42].

External influences from carers and social media played an important role in deprescribing. Asians were found to be more family-centric when compared to their Western counterparts, with many older people preferring their family members (e.g. adult children or spouses) as their carers [43]. These older persons particularly those who are unable to care for themselves may require primary support from their carers to manage their daily medications [44] and frequently prefer to make major medical decisions including deprescribing medications together with their carers [45, 46].

Technology advancements have contributed to an upsurge in the adoption of mobile devices with internet connectivity across all sociodemographic groups, including older persons [47]. In recent years, social media has evolved into a double-edged tool for finding healthcare information [48, 49]. The use of social media could assist patients in informing healthcare decision-making and removing the physical barriers that typically prevented access to healthcare support and resources [47]. However, patients may experience serious and even life-threatening consequences because of the unregulated nature of the information accessible on the internet [48, 49].

System challenges like time constraint, incomplete medical history and unavailability of medications could influence deprescribing decisions in doctors. Early-career primary care doctors were found to be particularly affected by time constraints in their clinic which could reduce their likelihood to initiate deprescribing [50]. Besides, some guidelines are lengthy and time-consuming to read [51]. To date, most deprescribing guidelines and interventions for older persons were developed in developed countries, which may not apply to countries with limited resources [52]. At present, Beer’s criteria and STOPP (Screening Tool of Older Persons’ Prescriptions) and START (Screening Tool to Alert to Right Treatment) are commonly used in Malaysia to identify PIMs in older persons [3, 53]. Doctors preferred continuous medical education to be added to their existing specialist training programme to increase their awareness and knowledge of deprescribing [54]. Some doctors requested the involvement of pharmacists to review medications [58]. Other doctors encouraged patients to bring a complete medication list or have medication charts in the clinic [55]. In Malaysia, the healthcare system provides medications to the public through two systems: the government-funded public sector and self-sufficient private sector [56]. However, the unavailability of medications in the public sector may be a result of inadequate funding, inaccurate demand forecasts, ineffective procurement and distribution of medications [57, 58]. Hence, some doctors were hesitant to deprescribe when the availability of medication differs from one healthcare facility to another, particularly in government-funded clinics in rural areas, where the budget allocated for medications can be limited [56].

The strength of this study was the application of the qualitative methodology to collect rich, in-depth information about the views of both doctors and older patients regarding the necessity and concerns of deprescribing medications in older persons. Primary care trainees were selected as our target population as they are our future family medicine specialists. It is therefore crucial to incorporate deprescribing into their specialist training given that our population is ageing. This study found that trainees lacked knowledge and expertise in deprescribing medication in older persons. Hence, training bodies should include deprescribing and appropriate prescribing as part of the core curriculum in the family medicine training programme to enhance trainees’ knowledge about it. One of the limitations was that no family medicine specialist was interviewed, and their views regarding deprescribing in older persons could have been different from the primary care trainees, given their expertise and professional experiences [33]. Hence, future studies on deprescribing in older persons should also explore family medicine specialists’ beliefs and attitudes toward deprescribing for a more holistic patient care and implementation strategy. Additionally, this study was conducted in a primary care clinic in a teaching hospital in which primary care trainees were constantly guided by specialists. This could limit the transferability of the findings.

## Conclusion

This study found that deprescribing was deemed necessary by both patients and doctors when there was a reason to do so. However, both doctors’ and patients’ had some fears about the consequences of deprescribing. The primary care trainees who are early-career doctors felt compelled to continue medications that were initiated by another specialist. Factors that influenced deprescribing were doctor-patient rapport, health literacy among patients, external influences from carers and social media, and system challenges. Future studies should also include carers’ and other healthcare providers’ views regarding the deprescribing of medications in older people so that a more holistic view regarding challenges in deprescribing medications in older persons can be obtained.

## Electronic supplementary material

Below is the link to the electronic supplementary material.

**Additional file 1** Topic guide

## Data Availability

The datasets generated and analyzed during the current study may be available from the corresponding author on reasonable request.

## Abbreviations

FGD: Focus group discussion
IDI: In-depth interview
IQR: Interquartile range
