# Supplementary material for: Weighing the necessities and concerns of deprescribing among older ambulatory patients and primary care trainees: a qualitative study
Source: BMC Prim Care. 2023 Jun 30;24:136. doi: 10.1186/s12875-023-02084-8 (PMC10311750; doi:10.1186/s12875-023-02084-8)
Supplement: Supplementary file 1 — Additional file 1 Topic guide [file 12875_2023_2084_MOESM1_ESM.docx]

**Additional file 1: Topic guide**

| Patients | Doctors |
| --- | --- |
| - What do you understand by the term ‘deprescribing’ or cutting down on the number of medication(s) you are currently taking? | - What do you understand by the term ‘deprescribing’ |
| - Have you ever considered cutting down on the number of medication(s) that you are currently taking?   - Are you having any problems with your medication(s)? - Do you talk to your doctor about cutting down your medication(s)? | - Would you deprescribe for your patients? - How would you deprescribe? - Do you talk to your patients about deprescribing? |
| - What would prevent you from cutting down on the number of medication(s) you are currently taking? | - What would prevent you from deprescribing? |
| - What would encourage you to cut down on the number of medication(s) you are currently taking? | - What would encourage you to deprescribe? |
